# Association of State Laws Regarding Snacks in US Schools With Students' Consumption of Solid Fats and Added Sugars

**DOI:** 10.1001/jamanetworkopen.2019.18436

**Published:** 2020-01-15

**Authors:** Lindsey Turner, Julien Leider, Elizabeth Piekarz-Porter, Jamie F. Chriqui

**Affiliations:** 1 College of Education, Boise State University, Boise, Idaho; 2 Institute for Health Research and Policy, University of Illinois at Chicago, Chicago; 3 Division of Health Policy and Administration, School of Public Health, University of Illinois at Chicago, Chicago

## Abstract

**Importance:**

The Smart Snacks in School standards (hereafter, *Smart Snacks*) were issued in 2013 with the aim of improving students’ dietary intake behaviors. Goals of Smart Snacks included reducing total energy intake, consumption of solid fats and added sugars, and sodium intake. Smart Snacks standards were required to be implemented by the start of the 2014 to 2015 school year at all US schools participating in federal child nutrition programs.

**Objective:**

To examine the association of state laws that specifically direct schools to implement Smart Snacks with student dietary consumption outcomes.

**Design, Setting, and Participants:**

This cross-sectional study used nationally representative data collected in the 2014 to 2015 school year as part of the School Nutrition and Meal Cost Study. Students in grades 1 through 12 (ages approximately 6-18 years) were randomly selected from 310 public schools in 30 US states and the District of Columbia. Analytic weights were applied and all percentages reported are weighted. Analyses were conducted from March 1, 2018, to December 12, 2019.

**Exposures:**

State laws requiring schools to implement Smart Snacks.

**Main Outcomes and Measures:**

A 24-hour recall was used to assess student dietary intake as daily kilocalories consumed as (a) total energy, (b) solid fats and added sugars combined, (c) solid fats, or (d) added sugars. Milligrams of daily sodium consumption were also computed.

**Results:**

Among 1959 students (mean [SD] age, 11.9 [3.5] years; 1014 [50.9%] boys), 420 students (22.5%) attended school in a state with Smart Snacks laws, and 528 students (26.1%) consumed snacks obtained at school. In covariate-adjusted models, total energy intake did not vary based on state law. Adjusted mean daily kilocalories from solid fats and added sugars was significantly lower among students in states with laws (508.7 [95% CI, 463.0 to 554.4] kcal) than among students in states without laws (562.5 [95% CI, 534.3 to 590.8] kcal; difference, −53.9 [95% CI, −104.5 to −3.2] kcal; *P* = .04). Consumption of sodium did not differ by state law. Kilocalories from solid fats contributed more to the difference than kilocalories from added sugars (−37.7 [95% CI −62.8 to −12.6] kcal vs −16.2 [95% CI, −51.3 to 19.0] kcal).

**Conclusions and Relevance:**

These findings suggest that students in states with laws requiring schools to implement Smart Snacks had better dietary intake than students in states without laws, consuming a mean of 53.9 fewer kilocalories from solid fats and added sugars per day, after adjusting for covariates. State-level policy mechanisms may support schools’ implementation of federal standards in ways that are associated with healthier diets among children and adolescents.

## Key Points

**Question:**

Are state laws requiring schools to implement federal Smart Snacks in School standards associated with student dietary consumption?

**Findings:**

In this cross-sectional study with a nationally representative sample of 1959 students in grades 1 through 12, students who attended schools in states with laws requiring the implementation of Smart Snacks in School standards consumed an adjusted mean of 53.9 fewer kcal from solid fats and added sugars per day than did students in states with no such laws, a statistically significant difference.

**Meaning:**

These findings suggest that state laws may support the implementation of federal standards, with significant implications for student dietary behaviors.

## Teaser

This cross-sectional study examines the association of state laws for implementation of the Smart Snacks in School with students’ dietary intake behaviors.

## Introduction

Dietary intake is the primary modifiable behavioral risk factor associated with morbidity and mortality among people in the United States, with unhealthful dietary habits associated with cardiovascular disease, stroke, diabetes, and obesity.^1,2^ The Dietary Guidelines for Americans provide science-based advice to reduce these risks through optimal diets. The 2010 guidelines^3^ recommend that children, adolescents, and adults limit their intake of empty calories, which are commonly obtained by consumption of foods and beverages that contain solid fats and added sugars.^4^ Solid fats and added sugars are characterized as empty calories^5^ because they do not provide essential nutrients, they displace the consumption of other nutrient-dense foods and beverages, and they increase overall energy intake.^3,5,6^ Schools are locations that have contributed substantially to the consumption of empty calories by children and adolescents.^7,8^

Large-scale studies conducted in the early 2000s reported that sugar-sweetened beverages, other sugary foods, and snacks high in fat and sodium were common in schools across the United States^9,10,11^ and significantly affected students’ consumption of empty calories. As directed by the Healthy, Hunger-Free Kids Act of 2010,^12^ the US Department of Agriculture (USDA) issued revised standards for school meals in 2012 and new standards for all foods and beverages sold in other locations at schools in 2013.^13^ These standards aligned with the Dietary Guidelines for Americans^3^ and science-based recommendations.^14^ The latter, named the Smart Snacks in School standards (hereafter, *Smart Snacks*), aimed to increase the availability and consumption of healthful options such as fruit, vegetables, whole grain foods, and low-fat dairy and to reduce availability and consumption of high-calorie items with high amounts of fat, added sugar, and sodium. Smart Snacks was required to be implemented in schools by July 1, 2014.^13^

Prior to 2014, some states, districts, and schools had already addressed the nutritional quality of foods and beverages sold in schools outside of school meals. The number of states with policies addressing nutrition standards for such foods and beverages increased between the 2006 to 2007 school year and the 2014 to 2015 school year, by which point 50% of states had strong laws and 13% of states had weak laws that established some type of nutrition standards.^15^ A systematic review in 2014^16^ found that such policies at the state or district level were associated with reduced availability of unhealthful foods and beverages sold outside of school meals (ie, in school stores or snack bars, vending machines, and à la carte lines).

Although all schools participating in USDA child nutrition programs are required to comply with Smart Snacks, state laws may further facilitate compliance with national policy, particularly during the early phases of implementation. This is an important question for understanding the mechanisms of policy implementation, but it has not been addressed empirically, to our knowledge. At the start of the 2014 to 2015 school year, 9 states had laws requiring compliance with Smart Snacks. Given that national implementation of Smart Snacks was slow and many schools found it challenging to fully implement these policy changes,^17^ having a state law may have facilitated implementation of systemic changes in school food and beverage environments.

This cross-sectional study examined associations of state laws requiring Smart Snacks implementation with student dietary outcomes in the 2014 to 2015 school year. The hypothesis was that such laws would be associated with better dietary outcomes among students attending schools in those states.

## Methods

This study combined student-level data collected by the nationally representative School Nutrition and Meal Cost Study (SNMCS)^18^ with corresponding state laws collected and coded by the National Wellness Policy Study (NWPS). A brief description of SNMCS data collection is provided here, with extensive details available elsewhere.^18^ The SNMCS protocol and instruments were reviewed and approved by the US Office of Management and Budget. Student assent and parental informed consent were obtained for the diet interview. Nonidentifiable data were provided to the authors by Mathematica Policy Research, which was contracted to conduct the SNMCS for the USDA. Per the Common Rule, the collection of data on state laws is not considered to be human subjects research, so ethics board approval was not required. The study is reported following the Strengthening the Reporting of Observational Studies in Epidemiology (STROBE) reporting guideline.

### Student-Level Data

The SNMCS was commissioned by the USDA to assess practices in a nationally representative sample of public schools serving kindergarten through 12th grade during the 2014 to 2015 school year. The universe for sampling included public school food authorities and public noncharter schools participating in the National School Lunch Program. A stratified 2-stage sampling approach was used, with 3 groups of school food authorities selected using probability proportional to size sampling. Within 1 of those school food authority groups, a sample of 310 schools was recruited; thereafter, students were randomly selected and recruited from those schools for interviews. Dietary interviews were conducted with 2165 respondents in grades 1 through 12 (63.6% weighted response rate).^19^ Children in kindergarten and prekindergarten were excluded from the study because of concerns about their ability to provide accurate dietary recalls.

#### Student Dietary Data Collection

As detailed in the SNMCS report,^19^ collection of student dietary data used a 24-hour dietary recall with the USDA’s Dietary Intake Data System.^20^ The computer-assisted Automated Multiple-Pass Method was used to collect information about each student’s dietary intake. Students in middle school (typically grades 6-8, including students ages approximately 11-14 years) and high school (typically grades 9-12, including students ages approximately 14-18 years) completed the interview independently, and students in elementary school (typically grades 1-5, including students ages approximately 6-11 years) had parental assistance. The USDA’s Post-Interview Processing System, combined with Survey Net,^19,20^ was used to code types and amounts of foods and beverages consumed, from which nutrient characteristics were computed. The analyses used information about total daily energy intake (in kilocalories), as well as daily energy intake from solid fats and added sugars and sodium intake. In the data coding process, SNMCS indicated whether students had consumed any snacks obtained at schools (eg, purchased from sales venues, given by teachers, or at parties; coded as yes or no).

#### Student Demographic Characteristics

The restricted-use data set supplied for this study included student demographic characteristics, which were used as contextual covariates in the analyses. Demographic variables included student sex (boys or girls), grade in school, and self- or parent-reported race/ethnicity. Additional demographic data for students were based on the school or district at which they were enrolled.^21,22,23^ These included region, school urbanicity, school size, racial/ethnic composition of the district’s students, and socioeconomic composition.

### State-Level Data

The NWPS is the largest nationwide evaluation of congressionally mandated school district wellness policies^24^ and state laws for 50 states and the District of Columbia^15^ (collectively referred to as *states*). Codified state statutes and administrative regulations for each state were compiled using subscription-based services, Lexis Advance (LexisNexis) and WestlawNext (Thomson Reuters). Boolean keyword searches and reviews of the indices or tables of contents of the codified laws for each state were conducted by trained attorneys and legal researchers using the state law databases from each service. State laws were defined to include the codified laws as well as any state health or nutrition education standards incorporated by reference into the codified law. Laws were deemed relevant if they were effective as of September 2, 2014, a proxy for the beginning of the 2014 to 2015 school year. The existence of state laws was verified against public sources.^25,26^

State laws were reviewed and verified by 2 members of the NWPS team (including E.P.-P.), then coded by 2 trained analysts (including E.P.-P.). Coding used a wellness policy coding scheme^27^ that was modified by NWPS to capture new Smart Snacks provisions. State laws were coded for whether they were definitively required; encouraged, suggested, or required with exceptions; or did not address compliance with Smart Snacks. Strong policy provisions definitively required implementation and met Smart Snacks standards^28,29^ if they included language such as *shall*, *must*, *will*, *require*, *comply*, and *enforce*. Laws were coded at elementary school (grades 1-5), middle school (grades 6-8), and high school (grades 9-12) levels. Locations of sale included 4 venues: vending machines, school stores or snack bars, à la carte, and fundraisers. Analyses used a dichotomous variable indicating whether state law met Smart Snacks standards in all 4 venues.

### Statistical Analysis

The SNMCS student-level data and state law data were linked using state geocodes by Mathematica Policy Research (Mathematica) and returned to the NWPS for analyses. Grade level–specific state law coding was linked to schools of the corresponding grade level. Analyses were conducted from March 1, 2018, to December 12, 2019, with Stata statistical software version 13 (StataCorp) using the *svy* command to account for the sampling design. Analytic weights were used; thus numbers of students are unadjusted, and all percentages given are weighted. Descriptive statistics for demographic variables were computed to examine characteristics of students. Descriptive statistics for student dietary intake were examined for all students and for the subset of students who consumed snacks at school. A series of multivariable linear regression models were computed to examine dietary outcomes of interest: (a) total daily energy intake in kilocalories, (b) kilocalories from solid fats and added sugars combined, (c) kilocalories from solid fats alone, (d) kilocalories from added sugars alone, and (e) sodium intake in milligrams. A multivariable logistic regression model was computed to examine differences between students who consumed snacks at school vs those who did not. After computing models for all students, regression models were recomputed for the subset of students who consumed snacks at school. Each model included state law as a key variable while controlling for contextual covariates of student, school, and district demographic characteristics. A 2-tailed a priori α level of .05 was used for significance tests, considering the coefficients for state law in the multivariable regression models. The adjusted margins for state law were examined, which represent the mean value of the outcome at each level while accounting for all other covariates.

## Results

Among 2165 students who completed the 24-hour dietary recall, 206 students had missing data for some demographic variables, reducing the analytical sample to 1959 students (mean [SD] age, 11.9 [3.5] years; 1014 [50.9%] boys) from 290 schools (Table 1). Descriptive statistics presented here may differ from the SNMCS report^19^ because of this restriction on the analytic sample. Among 1959 students sampled, 420 students (22.5%) attended school in a state with Smart Snack laws, and 528 students (26.1%) reported consuming snacks obtained at school on the day of reporting. Students attended schools in 30 states and the District of Columbia. Among these states, 7 had a Smart Snacks law (Arkansas, District of Columbia, Florida, Georgia, Illinois, Mississippi, and Utah). Students consumed a mean total of 1982.2 (95% CI, 1919.2 to 2045.2) kcal per day (Table 2), but as expected there were significant differences by demographic characteristics, such as grade in school and sex (Table 3). Few school-level characteristics were associated with students’ daily energy intake. State laws regarding Smart Snacks were not significantly associated with total energy intake among the full sample or among students who consumed snacks at school. Students attending schools in states with laws consumed an adjusted mean of 508.7 (95% CI, 463.0 to 554.4) kcal from solid fats and added sugars, which comprised 25.7% of their total daily energy intake, whereas students in states without laws consumed an adjusted mean of 562.5 (95% CI, 534.3 to 590.8) kcal, which comprised 28.4% of their total daily energy intake, a difference of −53.9 (95% CI, −104.5 to −3.2) kcal (*P* = .04) (Table 4). Calories from solid fats contributed more to the difference than calories from added sugars (−37.7 [95 CI, −62.8 to −12.6] kcal vs −16.2 [95% CI, −51.3 to 19.0] kcal). The pattern of results among students who consumed snacks at school was similar, but the difference was not statistically significant. Additional analyses examined the outcomes of consumption of solid fats alone, added sugars alone, and sodium (Table 5; eTables 1, 2, and 3 in the Supplement). Among all students, students in states with laws compared with those in states without laws had lower adjusted mean total daily consumption of solid fats (257.6 [95% CI, 237.1 to 278.1] kcal vs 295.3 [95% CI, 280.5 to 310.2] kcal; *P* = .004), but not added sugars or sodium. Results were similar for students who consumed snacks at school. Students who consumed snacks at school in states with laws consumed an adjusted mean of 247.1 (95% CI, 208.2 to 286.0) kcal from solid fats, whereas those in states without laws consumed an adjusted mean of 300.6 (95% CI, 272.1 to 329.1) kcal from solid fats, a difference of −53.5 (95% CI, −97.0 to −10.0) kcal (*P* = .02). To consider whether laws were associated with students’ at-school consumption behaviors, we used multivariable logistic regression to estimate snack consumption (eTable 4 in the Supplement). Similar percentages of students consumed snacks at school in states with laws (adjusted prevalence, 23.3% [95% CI, 18.3% to 28.4%]) as did in states without laws (adjusted prevalence, 26.9% [95% CI, 23.8% to 30.0%]; *P* = .25).

**Table 1.** Student-, School-, and District-Level Characteristics of Sample

Characteristic	Students, No. (%) (N = 1959)^a^
**Student Level**	
Grade^b^	
1	146 (8.7)
2	149 (9.6)
3	146 (10.0)
4	136 (8.7)
5	138 (7.9)
6	191 (7.2)
7	220 (7.5)
8	228 (7.2)
9	174 (9.5)
10	148 (8.1)
11	160 (9.2)
12	123 (6.5)
Sex	
Boys	1014 (50.9)
Girls	945 (49.1)
Race/ethnicity	
Non-Hispanic white	953 (52.6)
Non-Hispanic black	267 (13.3)
Hispanic	551 (25.5)
Other or multiracial	188 (8.7)
**School Level**	
Urbanicity	
Urban	436 (23.5)
Suburban	1019 (49.3)
Rural	504 (27.2)
Size, No. of students	
≥1000	561 (31.0)
500-999	878 (45.1)
<500	520 (23.9)
**District Level**	
Racial/ethnic composition of students	
≥66% White	650 (37.3)
≥50% Black	83 (4.6)
≥50% Hispanic	465 (17.5)
Other	761 (40.6)
District-level child poverty rate	
<20%	996 (67.2)
≥20%	963 (32.8)
Region	
West	486 (17.5)
Midwest	457 (24.6)
South	826 (44.4)
Northeast	190 (13.5)
State law requires schools to meet Smart Snacks in all venues	
No	1539 (77.5)
Yes	420 (22.5)

^a^ No. is unweighted, percentage is survey-weighted.

^b^ Approximate student ages by grade: grade 1, 6 to 7 years; grade 2, 7 to 8 years; grade 3, 8 to 9 years; grade 4, 9 to 10 years; grade 5, 10 to 11 years; grade 6, 11 to 12 years; grade 7, 12 to 13 years; grade 8, 13 to 14 years; grade 9, 14 to 15 years; grade 10, 15 to 16 years; grade 11, 16 to 17 years; and grade 12, 17 to 18 years.

**Table 2.** Mean Daily Energy Intake and Sodium Intake

Intake	Mean (95% CI), kcal	
	All Students (N = 1959)	Students Who Consumed Snacks Obtained at School (n = 528)
Total daily energy	1982.2 (1919.2 to 2045.2)	1997.1 (1894.0 to 2100.2)
Solid fats and added sugars	550.4 (524.5 to 576.4)	568.3 (526.0 to 610.7)
Solid fats only	286.9 (273.9 to 299.8)	289.1 (262.6 to 315.6)
Added sugars only	263.6 (246.6 to 280.6)	279.2 (257.4 to 301.1)
Sodium, mg	3165.8 (3057.7 to 3273.9)	3163.0 (2977.6 to 3348.4)

**Table 3.** Multivariable Linear Regression Model Examining the Association of State-Level Smart Snacks Policy With Students’ Total Daily Energy Intake

Variable	All Students (N = 1959)			Students Who Consumed Snacks Obtained at School (n = 528)		
	Coefficient (95% CI), kcal	*P* Value	Adjusted Mean (95% CI), kcal	Coefficient (95% CI), kcal	*P* Value	Adjusted Mean (95% CI), kcal
State law requires Smart Snacks standards to be followed in all school venues						
No	0 [Reference]	NA	1983.5 (1919.1 to 2047.9)	0 [Reference]	NA	2032.0 (1922.7 to 2141.4)
Yes	−5.77 (−145.65 to 134.10)	.94	1977.7 (1841.6 to 2113.9)	−162.64 (−334.30 to 9.03)	.06	1869.4 (1705.2 to 2033.6)
Student grade, per increased grade level	17.49 (1.22 to 33.75)	.04	NA	10.98 (−14.49 to 36.46)	.39	NA
Sex						
Boys	0 [Reference]	NA	2144.4 (2050.3 to 2238.4)	0 [Reference]	NA	2292.2 (2132.4 to 2452.0)
Girls	−330.10 (−437.06 to −223.14)	<.001	1814.3 (1750.2 to 1878.4)	−542.50 (−723.25 to −361.74)	<.001	1749.7 (1655.5 to 1843.9)
Race/ethnicity						
Non-Hispanic white	0 [Reference]	NA	1970.2 (1880.6 to 2059.9)	0 [Reference]	NA	2018.6 (1876.2 to 2160.9)
Non-Hispanic black	67.31 (−100.08 to 234.69)	.43	2037.5 (1884.9 to 2190.2)	15.22 (−183.67 to 214.10)	.88	2033.8 (1886.1 to 2181.5)
Hispanic	19.08 (−111.42 to 149.57)	.77	1989.3 (1891.6 to 2087.0)	−20.64 (−347.57 to 306.29)	.90	1997.9 (1722.2 to 2273.6)
Other or multiracial	−20.94 (−179.65 to 137.76)	.79	1949.3 (1797.6 to 2101.0)	−230.07 (−518.12 to 57.97)	.12	1788.5 (1546.6 to 2030.4)
School urbanicity						
Urban	0 [Reference]	NA	2048.0 (1871.4 to 2224.5)	0 [Reference]	NA	2042.4 (1908.8 to 2176.0)
Suburban	−92.09 (−295.95 to 111.78)	.37	1955.9 (1883.1 to 2028.6)	−26.47 (−177.72 to 124.79)	.73	2015.9 (1872.9 to 2159.0)
Rural	−74.80 (−265.69 to 116.08)	.44	1973.2 (1866.2 to 2080.1)	−119.08 (−281.63 to 43.47)	.15	1923.3 (1791.3 to 2055.3)
School size, No. of students						
≥1000	0 [Reference]	NA	2067.1 (1957.9 to 2176.2)	0 [Reference]	NA	2094.4 (1907.3 to 2281.4)
500-999	−140.48 (−277.71 to −3.26)	.045	1926.6 (1845.8 to 2007.3)	−222.21 (−428.85 to −15.58)	.04	1872.2 (1756.7 to 1987.6)
<500	−90.05 (−209.71 to 29.61)	.14	1977.0 (1883.7 to 2070.3)	−20.14 (−279.84 to 239.56)	.88	2074.2 (1901.5 to 2246.9)
District-level racial/ethnic composition						
≥66% White	0 [Reference]	NA	2049.4 (1957.8 to 2141.1)	0 [Reference]	NA	2056.3 (1901.4 to 2211.2)
≥50% Black	−83.63 (−261.43 to 94.17)	.35	1965.8 (1801.7 to 2130.0)	10.25 (−351.80 to 372.30)	.96	2066.5 (1701.8 to 2431.2)
≥50% Hispanic	−65.73 (−255.08 to 123.61)	.49	1983.7 (1826.0 to 2141.5)	−27.85 (−228.60 to 172.90)	.78	2028.4 (1890.8 to 2166.1)
Other	−127.80 (−255.69 to 0.09)	.05	1921.6 (1834.0 to 2009.3)	−146.14 (−326.36 to 34.08)	.11	1910.1 (1792.9 to 2027.4)
District-level child poverty rate						
<20%	0 [Reference]	NA	1986.2 (1893.3 to 2079.0)	0 [Reference]	NA	1985.5 (1863.0 to 2108.0)
≥20%	−12.05 (−137.72 to 113.61)	.85	1974.1 (1903.2 to 2045.0)	35.29 (−113.93 to 184.51)	.64	2020.8 (1892.6 to 2149.1)
Region						
West	0 [Reference]	NA	2025.8 (1933.3 to 2118.3)	0 [Reference]	NA	1997.5 (1863.7 to 2131.2)
Midwest	52.90 (−83.87 to 189.68)	.45	2078.7 (1978.3 to 2179.1)	79.09 (−132.98 to 291.15)	.46	2076.5 (1912.3 to 2240.8)
South	−39.35 (−190.25 to 111.55)	.61	1986.5 (1894.5 to 2078.4)	51.14 (−130.12 to 232.40)	.58	2048.6 (1909.3 to 2187.9)
Northeast	−291.07 (−498.30 to −83.85)	.006	1734.7 (1531.8 to 1937.7)	−379.80 (−685.14 to −74.45)	.02	1617.7 (1318.6 to 1916.7)

Abbreviation: NA, not applicable.

**Table 4.** Multivariable Linear Regression Model Examining the Association of State-Level Smart Snacks Policy With Students’ Daily Energy Intake From Solid Fats and Added Sugars

Variable	All Students (N = 1959)			Students Who Consumed Snacks Obtained at School (n = 528)		
	Coefficient (95% CI), kcal	*P* Value	Adjusted Mean (95% CI), kcal	Coefficient (95% CI), kcal	*P* Value	Adjusted Mean (95% CI), kcal
State law requires Smart Snacks standards to be followed in all school venues						
No	0 [Reference]	NA	562.5 (534.3 to 590.8)	0 [Reference]	NA	582.0 (539.0 to 625.0)
Yes	−53.87 (−104.52 to −3.22)	.04	508.7 (463.0 to 554.4)	−63.53 (−137.93 to 10.86)	.09	518.5 (444.2 to 592.8)
Student grade, per increased grade level	4.76 (−3.00 to 12.51)	.23	NA	3.79 (−8.46 to 16.04)	.54	NA
Sex						
Boys	0 [Reference]	NA	593.6 (559.6 to 627.7)	0 [Reference]	NA	650.6 (589.1 to 712.0)
Girls	−87.90 (−123.58 to −52.23)	<.001	505.7 (478.0 to 533.4)	−151.11 (−216.69 to −85.53)	<.001	499.4 (458.8 to 540.1)
Race/ethnicity						
Non-Hispanic white	0 [Reference]	NA	553.0 (522.2 to 583.8)	0 [Reference]	NA	574.0 (514.5 to 633.5)
Non-Hispanic black	37.28 (−33.58 to 108.14)	.30	590.2 (519.5 to 661.0)	50.21 (−69.01 to 169.44)	.41	624.2 (521.3 to 727.1)
Hispanic	−20.99 (−73.86 to 31.89)	.43	532.0 (485.7 to 578.3)	−29.44 (−158.78 to 99.91)	.65	544.5 (438.9 to 650.1)
Other or multiracial	−24.38 (−94.69 to 45.93)	.49	528.6 (459.1 to 598.1)	−86.31 (−232.78 to 60.15)	.25	487.7 (370.1 to 605.3)
School urbanicity						
Urban	0 [Reference]	NA	600.3 (546.4 to 654.1)	0 [Reference]	NA	647.4 (589.1 to 705.6)
Suburban	−77.88 (−138.23 to −17.53)	.01	522.4 (493.0 to 551.8)	−102.92 (−163.38 to −42.47)	.001	544.4 (497.5 to 591.4)
Rural	−42.00 (−108.76 to 24.76)	.22	558.3 (510.8 to 605.7)	−106.06 (−184.09 to −28.04)	.008	541.3 (468.0 to 614.6)
School size, No. of students						
≥1000	0 [Reference]	NA	554.5 (513.5 to 595.5)	0 [Reference]	NA	603.5 (529.0 to 678.0)
500-999	−13.60 (−67.84 to 40.64)	.62	540.9 (503.7 to 578.2)	−82.62 (−164.31 to −0.94)	.047	520.9 (469.6 to 572.1)
<500	8.56 (−45.83 to 62.96)	.76	563.1 (522.1 to 604.1)	−2.79 (−115.49 to 109.91)	.96	600.7 (528.4 to 673.0)
District-level racial/ethnic composition						
≥66% White	0 [Reference]	NA	602.4 (562.5 to 642.3)	0 [Reference]	NA	611.9 (546.5 to 677.4)
≥50% Black	−62.17 (−149.31 to 24.98)	.16	540.2 (461.6 to 618.8)	−95.35 (−231.44 to 40.74)	.17	516.6 (391.6 to 641.6)
≥50% Hispanic	−90.19 (−171.99 to −8.40)	.03	512.2 (445.8 to 578.5)	−69.94 (−168.48 to 28.60)	.16	542.0 (464.2 to 619.8)
Other	−82.01 (−134.12 to −29.90)	.002	520.4 (486.0 to 554.7)	−67.88 (−147.48 to 11.72)	.09	544.0 (491.8 to 596.3)
District-level child poverty rate						
<20%	0 [Reference]	NA	551.5 (514.4 to 588.6)	0 [Reference]	NA	563.0 (505.8 to 620.1)
≥20%	−3.22 (−55.59 to 49.15)	.90	548.3 (515.4 to 581.1)	16.46 (−57.58 to 90.50)	.66	579.4 (530.1 to 628.8)
Region						
West	0 [Reference]	NA	558.9 (516.7 to 601.2)	0 [Reference]	NA	580.1 (528.2 to 632.1)
Midwest	19.40 (−35.13 to 73.93)	.48	578.3 (547.0 to 609.7)	46.42 (−30.98 to 123.83)	.24	626.6 (570.8 to 682.4)
South	5.68 (−62.88 to 74.24)	.87	564.6 (522.0 to 607.3)	−3.39 (−83.91 to 77.13)	.93	576.7 (516.1 to 637.4)
Northeast	−117.42 (−198.95 to −35.90)	.005	441.5 (363.0 to 520.0)	−187.83 (−300.95 to −74.70)	.001	392.3 (287.0 to 497.6)

Abbreviation: NA, not applicable.

**Table 5.** Covariate-Adjusted Student Dietary Outcomes by State-Level Smart Snacks Policy

Variable	All Students (N = 1959)				Students Who Consumed Snacks Obtained at School (n = 528)			
	Adjusted Mean (95% CI)		Difference	*P* Value	Adjusted Mean (95% CI)		Difference	*P* Value
	In States Without a Law	In States With a Law			In States Without a Law	In States With a Law		
Total daily energy intake, kcal	1983.5 (1919.1 to 2047.9)	1977.7 (1841.6 to 2113.9)	−5.8	.94	2032.0 (1922.7 to 2141.4)	1869.4 (1705.2 to 2033.6)	−162.6	.06
Solid fats and added sugars, kcal	562.5 (534.3 to 590.8)	508.7 (463.0 to 554.4)	−53.9	.04	582.0 (539.0 to 625.0)	518.5 (444.2 to 592.8)	−63.5	.09
Solid fats, kcal	295.3 (280.5 to 310.2)	257.6 (237.1 to 278.1)	−37.7	.004	300.6 (272.1 to 329.1)	247.1 (208.2 to 286.0)	−53.5	.02
Added sugars, kcal	267.2 (248.4 to 286.0)	251.1 (219.4 to 282.7)	−16.2	.36	281.4 (259.8 to 303.0)	271.4 (224.1 to 318.7)	−10.0	.67
Sodium, mg	3162.7 (3056.0 to 3269.5)	3176.3 (2927.8 to 3424.8)	13.5	.92	3223.3 (3006.6 to 3440.1)	2942.7 (2648.4 to 3237.0)	−280.6	.12

## Discussion

This cross-sectional study examining differences in the dietary intake of students in states subject to laws regarding Smart Snacks vs students in states without such laws found that some dietary outcomes differed by state law among the overall sample and among students who consumed snacks at school on the measurement day. Among all students, those in states with laws requiring schools to implement Smart Snacks consumed an adjusted mean of 53.9 fewer total kcal per day from solid fats and added sugars. More of this difference came from solid fats (37.7 kcal) than added sugars (16.2 kcal); when examined as separate outcomes, solid fat consumption differed significantly by state law, but consumption of added sugars did not. When considering the subsample of students who consumed snacks at school, while the reduction limited statistical power, a similar pattern was seen, although the difference was not statistically significant. Students who consumed snacks at school in states with laws consumed 53.5 fewer kcal from solid fats compared with students in states without laws. Differences in daily consumption of solid fats and added sugars combined and sodium were not statistically significant.

Policy approaches to improving the school food environment are hypothesized to work by improving the nutritional composition of foods and beverages sold at school, and thereby changing students’ dietary intake behaviors. However, another way they may affect behavior is by reducing students’ snacking behaviors—that is, decreasing the frequency of purchasing and consuming any snacks at school. We did not find that state laws were associated with differences in the percentages of students who consumed snacks at school; however, for students who consumed snacks at school, dietary outcomes were significantly different. This suggests that the association of policy with dietary outcomes works by changing the nutritional composition of items sold in school without an associated change in snacking behaviors—in other words, students may keep snacking, but when the snacks sold are more healthful, the dietary patterns of those students may improve.

The differences observed in students’ total dietary energy intake may seem modest, but a 54 kcal reduction in solid fats and added sugars is significant practically as well as statistically. Consumption of solid fats and added sugars adds between 500 to 1050 kcal to total daily energy intake for people in the United States,^3^ far higher than optimal levels: the 2015 to 2020 Dietary Guidelines reiterated the importance of limiting solid fats and added sugars consumption, recommending no more than 10% of kcal daily from sugar and no more than 10% from saturated fat for all age groups.^30^ Our findings, which used data from a nationally-representative sample of children and adolescents, suggest that more than 25% of daily energy intake was derived from solid fats and added sugars, with solid fats and added sugars consumption each over 10%. While the subset of students who consumed snacks at school also exceeded those recommendations, students in states with laws that address the nutritional standard of school snacks had significantly better dietary patterns. Studies have shown that over time, small changes in daily dietary intake can substantially improve health outcomes, including weight status and cardiovascular outcomes associated with consumption of solid fats and added sugars.^3,4^

### Limitations

This study has several limitations. National-level policy interventions, such as Smart Snacks, and state laws to support implementation are expected to improve the nutritional characteristics of foods and beverages sold to students on campus. Mediation analyses to examine the hypothesized associations could not be conducted because we did not have intermediate analytic weights for schools, which would be necessary for multilevel analyses. Furthermore, data were not available on the nutrient profiles of snacks sold at each school. As a result, these analyses do not explicitly demonstrate that policy changed the types of foods and beverages sold at the school that each student attended. Such analyses should be conducted in the future. Furthermore, the cross-sectional nature of these data does not allow for examination of changes in students’ dietary patterns. It is expected that policy changes would result in measurable changes in school-level food and beverage nutritional quality, as well as student-level dietary outcomes. Such changes cannot be examined with cross-sectional data; thus, these associations may not be causal or directional—third variables may explain the associations, such as other characteristics of the states with laws. However, we accounted for student demographic characteristics and other contextual characteristics. Furthermore, the states with laws are located in various regions of the United States and do not include states that have a history of state-level intervention to change school food environments. For example, California has a state nutrition policy, but we did not code it as having a Smart Snacks law because the state law was less stringent than Smart Snacks on some topics. The association of nutrition standards with dietary outcomes might be larger if a broader definition were used. The exposure variable was a state-level Smart Snacks law because our goal was to examine the potential for state-level policy actions to facilitate and support national-level policy intervention.

Although all schools participating in USDA child nutrition programs must comply with Smart Snacks, some states reinforced this requirement and were quicker to implement those standards. As we note elsewhere,^15^ states incorporated Smart Snacks in various ways. For example, state laws in Arkansas, Arizona, Florida, Iowa, and Mississippi adopted the full text of Smart Snacks or linked to the USDA website or another state-adopted policy that listed the full text of the standards. The District of Columbia, Georgia, Illinois, and Utah law instead provided a general reference to the federal rule without listing details for compliance.

Additionally, several points are noteworthy regarding the outcome. Only 1 day of dietary recall data were used, so although the sample of students was representative, their dietary intake on the measurement day represents only that day and may not be representative of each student’s overall dietary pattern owing to intraindividual variation in dietary intake. Response biases may have affected dietary self-report, and parental assistance for younger children may have biased responses. Regressions did not include a student-level measure of economic status, such as free or reduced-priced meal eligibility; students from wealthier families are more likely to be able to afford snacks, so the results for the sample of 528 students who consumed snacks at schools may include more students from wealthier families than the sample overall. Additionally, we recognize that there is not agreement among nutritional scientists that all solid fats and added sugars should be limited because some foods high in added sugars, such as some cereals and grain products, can contribute micronutrients to people’s diets.^4^ Nevertheless, the Dietary Guidelines focus on limiting energy intake from solid fats and added sugars and consuming nutrient-dense foods, such as lean meat, low-fat dairy, and fruits and vegetables; these are evidence-based strategies with scientific support.^3^

## Conclusions

In summary, our findings suggest that policy interventions, such as the Smart Snacks standards and state laws that support the implementation of these changes in schools, may be promising interventions for improving the dietary habits of children and adolescents. Owing to the significant negative health consequences associated with suboptimal diets, interventions to improve the dietary habits of people in the United States may be of significant value for the nation’s health.

## Notes

Supplement. **eTable 1.** Multivariable Linear Regression Model Examining the Association of State-Level Smart Snacks Policy With Students’ Daily Energy Intake From Solid Fats in Kilocalories **eTable 2.** Multivariable Linear Regression Model Examining the Association of State-Level Smart Snacks Policy With Students’ Daily Energy Intake From Added Sugars in Kilocalories **eTable 3.** Multivariable Linear Regression Model Examining the Association of State-Level Smart Snacks Policy With Students’ Daily Intake of Sodium in Milligrams **eTable 4.** Multivariable Logistic Regression Model Examining the Association of State-Level Smart Snacks Policy With Student Consumption of Snacks Obtained at School Among 1959 Students
