# Supplementary material for: Association of State Laws Regarding Snacks in US Schools With Students' Consumption of Solid Fats and Added Sugars
Source: JAMA Netw Open. 2020 Jan 15;3(1):e1918436. doi: 10.1001/jamanetworkopen.2019.18436 (PMC6991240; doi:10.1001/jamanetworkopen.2019.18436)
Supplement: Supplement. — eTable 1. Multivariable Linear Regression Model Examining the Association of State-Level Smart Snacks Policy With Students’ Daily Energy Intake From Solid Fats in Kilocalories eTable 2. Multivariable Linear Regression Model Examining the Association of State-Level Smart Snacks Policy With Students’ Daily Energy Intake From Added Sugars in Kilocalories eTable 3. Multivariable Linear Regression Model Examining the Association of State-Level Smart Snacks Policy With Students’ Daily Intake of Sodium in Milligrams eTable 4. Multivariable Logistic Regression Model Examining the Association of State-Level Smart Snacks Policy With Student Consumption of Snacks Obtained at School Among 1959 Students [file jamanetwopen-3-e1918436-s001.pdf]

Supplementary Online Content

Turner L, Leider J, Piekarz-Porter E, Chriqui JF. Association of state laws regarding snacks in US schools with students' consumption of solid fats and added sugars. *JAMA Network Open*. 2020;3(1):e1918436. doi:10.1001/jamanetworkopen.2019.18436

eTable 1. Multivariable Linear Regression Model Examining the Association of State-Level Smart Snacks Policy With Students' Daily Energy Intake From Solid Fats in Kilocalories

eTable 2. Multivariable Linear Regression Model Examining the Association of State-Level Smart Snacks Policy With Students' Daily Energy Intake From Added Sugars in Kilocalories

eTable 3. Multivariable Linear Regression Model Examining the Association of State-Level Smart Snacks Policy With Students' Daily Intake of Sodium in Milligrams

eTable 4. Multivariable Logistic Regression Model Examining the Association of State-Level Smart Snacks Policy With Student Consumption of Snacks Obtained at School Among 1959 Students

This supplementary material has been provided by the authors to give readers additional information about their work.

© 2020 Turner L et al. *JAMA Network Open.*

eTable 1. Multivariable Linear Regression Model Examining the Association of State-Level Smart Snacks Policy With Students' Daily Energy Intake From Solid Fats in Kilocalories

Variable	All Students (n = 1959)			95% CI	Students who Consumed Snacks Purchased at School (n = 528)			
	Coefficient (95% CI)	P Value	Adjusted Mean		Coefficient (95% CI)	P Value	Adjusted Mean	
State Law Requires Smart Snacks Standards to Be Followed in All School Venues								
No	0 [Reference]	--	295.3	280.5, 310.2	0 [Reference]	--	300.6	272.1, 329.1
Yes	-37.70 (-62.78, -12.62)	.004	257.6	237.1, 278.1	-53.50 (-97.03, -9.97)	.02	247.1	208.2, 286.0
Student Grade (continuous)	2.89 (-1.44, 7.21)	.19	--		2.53 (-4.77, 9.83)	.49	--	
Student Gender								
Male	0 [Reference]	--	312.7	295.7, 329.8	0 [Reference]	--	346.3	306.1, 386.6
Female	-52.69 (-71.87, -33.50)	<.001	260.1	245.4, 274.7	-105.18 (-147.42, -62.95)	<.001	241.1	217.5, 264.7
Student Race/Ethnicity								
White, non-Hispanic	0 [Reference]	--	290.5	274.2, 306.7	0 [Reference]	--	299.0	258.9, 339.0
Black, non-Hispanic	-0.88 (-39.88, 38.13)	.96	289.6	252.0, 327.1	1.20 (-79.18, 81.58)	.98	300.2	236.7, 363.6
Hispanic	-12.68 (-43.87, 18.51)	.42	277.8	251.2, 304.4	-22.30 (-103.22, 58.61)	.59	276.7	209.9, 343.4
Other	-3.03 (-44.80, 38.74)	.89	287.4	246.6, 328.3	-55.31 (-136.96, 26.33)	.18	243.7	182.5, 304.8
School Urbanicity								
Urban	0 [Reference]	--	303.5	272.4, 334.6	0 [Reference]	--	315.1	275.5, 354.6
Suburban	-27.30 (-65.23, 10.63)	.16	276.2	258.6, 293.8	-25.75 (-66.30, 14.81)	.21	289.3	258.4, 320.3
Rural	-11.71 (-47.52, 24.10)	.52	291.8	269.2, 314.4	-49.27 (-99.02, 0.47)	.052	265.8	223.1, 308.4
School Size								
Large (≥1000 students)	0 [Reference]	--	286.3	264.5, 308.1	0 [Reference]	--	303.3	252.4, 354.1
Medium (500 to 999 students)	-3.21 (-34.03, 27.61)	.84	283.1	263.8, 302.4	-36.51 (-95.59, 22.57)	.22	266.8	233.0, 300.5
Small (< 500 students)	8.26 (-20.81, 37.33)	.57	294.6	270.5, 318.7	5.07 (-55.97, 66.12)	.87	308.3	271.3, 345.3
Race/Ethnicity (District)								
≥66% White	0 [Reference]	--	291.8	272.4, 311.3	0 [Reference]	--	281.8	237.3, 326.4
≥50% Black	4.12 (-46.44, 54.68)	.87	296.0	247.7, 344.3	-3.94 (-93.84, 85.96)	.93	277.9	198.5, 357.3
≥50% Hispanic	-5.37 (-55.79, 45.06)	.83	286.5	243.6, 329.4	25.27 (-38.73, 89.27)	.44	307.1	260.9, 353.4
Other	-10.47 (-36.30, 15.35)	.42	281.4	264.6, 298.1	7.38 (-46.65, 61.41)	.79	289.2	257.3, 321.2
Child Poverty Rate (District)								
<20%	0 [Reference]	--	289.0	269.5, 308.6	0 [Reference]	--	292.0	256.6, 327.4
≥20%	-6.63 (-36.77, 23.51)	.66	282.4	264.1, 300.7	-8.99 (-58.32, 40.34)	.72	283.0	248.2, 317.9
Region								
West	0 [Reference]	--	298.4	274.1, 322.7	0 [Reference]	--	288.4	248.0, 328.7
Midwest	10.20 (-21.83, 42.23)	.53	308.6	291.8, 325.4	41.15 (-13.56, 95.86)	.14	329.5	293.0, 366.0

© 2020 Turner L et al. *JAMA Network Open.*

South	-11.23 (-52.63, 30.16)	.59	287.2	263.7, 310.6	-1.92 (-59.55, 55.72)	.95	286.4	246.8, 326.1
Northeast	-67.30 (-110.47, -24.13)	.003	231.1	193.2, 269.0	-75.12 (-150.74, 0.50)	.052	213.2	148.3, 278.2

© 2020 Turner L et al. *JAMA Network Open.*

eTable 2. Multivariable Linear Regression Model Examining the Association of State-Level Smart Snacks Policy With Students' Daily Energy Intake From Added Sugars in Kilocalories

Variable	All Students (n = 1959)				Students who Consumed Snacks Obtained at School (n = 528)			
	Coefficient (95% CI)	P Value	Adjusted Mean	95% CI	Coefficient (95% CI)	P Value	Adjusted Mean	95% CI
State Law Requires Smart Snacks Standards to Be Followed in All School Venues								
No	0 [Reference]	--	267.2	248.4, 286.0	0 [Reference]	--	281.4	259.8, 303.0
Yes	-16.17 (-51.28, 18.95)	.36	251.1	219.4, 282.7	-10.04 (-56.92, 36.85)	.67	271.4	224.1, 318.7
Student Grade (continuous)	1.87 (-2.63, 6.37)	.41	--		1.25 (-6.73, 9.24)	.76	--	
Student Gender								
Male	0 [Reference]	--	280.9	258.3, 303.5	0 [Reference]	--	304.2	273.6, 334.8
Female	-35.22 (-56.92, -13.52)	.002	245.7	228.7, 262.6	-45.93 (-80.19, -11.67)	.009	258.3	234.3, 282.3
Student Race/Ethnicity								
White, non-Hispanic	0 [Reference]	--	262.5	242.7, 282.4	0 [Reference]	--	275.0	246.3, 303.7
Black, non-Hispanic	38.16 (-1.24, 77.55)	.06	300.7	261.4, 339.9	49.02 (0.05, 97.99)	.050	324.0	275.5, 372.5
Hispanic	-8.31 (-38.56, 21.93)	.59	254.2	227.2, 281.2	-7.13 (-67.43, 53.16)	.82	267.9	220.7, 315.0
Other	-21.35 (-60.73, 18.03)	.29	241.2	201.4, 280.9	-31.00 (-106.20, 44.20)	.42	244.0	177.2, 310.8
School Urbanicity								
Urban	0 [Reference]	--	296.8	267.3, 326.2	0 [Reference]	--	332.3	297.0, 367.6
Suburban	-50.57 (-81.13, -20.02)	.001	246.2	227.4, 264.9	-77.17 (-116.39, -37.96)	<.001	255.1	230.6, 279.6
Rural	-30.28 (-71.16, 10.59)	.15	266.5	233.9, 299.1	-56.79 (-107.13, -6.45)	.03	275.5	233.2, 317.8
School Size								
Large (≥1000 students)	0 [Reference]	--	268.2	238.8, 297.6	0 [Reference]	--	300.2	259.7, 340.8
Medium (500 to 999 students)	-10.39 (-42.32, 21.54)	.52	257.8	235.0, 280.6	-46.11 (-90.86, -1.36)	.04	254.1	227.0, 281.3
Small (< 500 students)	0.30 (-39.92, 40.53)	.99	268.5	244.4, 292.6	-7.86 (-84.18, 68.45)	.84	292.4	244.1, 340.6
Race/Ethnicity (District)								
≥66% White	0 [Reference]	--	310.5	280.8, 340.2	0 [Reference]	--	330.1	297.1, 363.0
≥50% Black	-66.29 (-116.43, -16.15)	.01	244.2	201.8, 286.6	-91.41 (-161.69, -21.12)	.01	238.7	172.8, 304.6
≥50% Hispanic	-84.82 (-130.14, -39.51)	<.001	225.7	195.6, 255.8	-95.20 (-156.03, -34.37)	.002	234.9	185.6, 284.2
Other	-71.54 (-109.09, -33.98)	<.001	239.0	217.0, 261.0	-75.26 (-117.77, -32.76)	.001	254.8	223.7, 285.9
Child Poverty Rate (District)								
<20%	0 [Reference]	--	262.5	240.3, 284.7	0 [Reference]	--	270.9	242.4, 299.4
≥20%	3.40 (-23.97, 30.77)	.81	265.9	246.5, 285.3	25.45 (-15.46, 66.36)	.22	296.4	266.6, 326.2
Region								
West	0 [Reference]	--	260.6	235.4, 285.7	0 [Reference]	--	291.8	259.9, 323.6
Midwest	9.21 (-25.59, 44.00)	.60	269.8	245.9, 293.6	5.28 (-40.81, 51.36)	.82	297.1	262.2, 331.9
South	16.91 (-17.99, 51.82)	.34	277.5	251.8, 303.2	-1.47 (-47.08, 44.15)	.95	290.3	260.2, 320.4
Northeast	-50.13 (-102.23, 1.98)	.06	210.4	161.3, 259.6	-112.70 (-168.44, -56.96)	<.001	179.1	128.7, 229.4

© 2020 Turner L et al. *JAMA Network Open.*

eTable 3. Multivariable Linear Regression Model Examining the Association of State-Level Smart Snacks Policy With Students' Daily Intake of Sodium in Milligrams

Variable	All Students (n = 1959)				Students who Consumed Snacks Obtained at School (n = 528)			
	**Coefficient (95% CI)**	**P Value**	**Adjusted Mean**	**95% CI**	**Coefficient (95% CI)**	**P Value**	**Adjusted Mean**	**95% CI**
State Law Requires Smart Snacks Standards to Be Followed in All School Venues								
No	0 [Reference]	--	3162.7	3056.0, 3269.5	0 [Reference]	--	3223.3	3006.6, 3440.1
Yes	13.55 (-238.71, 265.81)	.92	3176.3	2927.8, 3424.8	-280.60 (-634.20, 72.99)	.12	2942.7	2648.4, 3237.0
Student Grade (continuous)	45.36 (19.14, 71.58)	.001	--		46.24 (-10.86, 103.33)	.11	--	
Student Gender								
Male	0 [Reference]	--	3453.5	3297.1, 3609.9	0 [Reference]	--	3636.9	3337.3, 3936.6
Female	-585.56 (-764.04, -407.09)	<.001	2867.9	2754.8, 2981.0	-871.15 (-1206.22, -536.08)	<.001	2765.8	2591.9, 2939.7
Student Race/Ethnicity								
White, non-Hispanic	0 [Reference]	--	3106.9	2948.1, 3265.6	0 [Reference]	--	3154.7	2883.3, 3426.0
Black, non-Hispanic	102.52 (-186.59, 391.63)	.48	3209.4	2968.9, 3449.9	3.83 (-435.40, 443.05)	.99	3158.5	2843.4, 3473.5
Hispanic	121.85 (-113.02, 356.71)	.31	3228.7	3058.2, 3399.3	-87.23 (-575.70, 401.23)	.72	3067.4	2684.9, 3449.9
Other	164.08 (-181.47, 509.62)	.35	3271.0	2938.6, 3603.3	350.48 (-629.38, 1330.34)	.48	3505.1	2543.5, 4466.8
School Urbanicity								
Urban	0 [Reference]	--	3213.8	2914.2, 3513.3	0 [Reference]	--	3105.1	2836.5, 3373.6
Suburban	-102.26 (-453.36, 248.84)	.57	3111.5	2980.1, 3242.9	79.71 (-330.56, 489.99)	.70	3184.8	2890.1, 3479.4
Rural	9.06 (-343.47, 361.59)	.96	3222.8	3010.2, 3435.5	70.14 (-396.44, 536.73)	.77	3175.2	2705.3, 3645.1
School Size								
Large (≥1000 students)	0 [Reference]	--	3368.0	3168.6, 3567.3	0 [Reference]	--	3393.9	2954.5, 3833.2
Medium (500 to 999 students)	-303.83 (-553.41, -54.26)	.02	3064.2	2925.2, 3203.2	-425.93 (-911.23, 59.38)	.09	2967.9	2748.2, 3187.7
Small (< 500 students)	-272.92 (-521.95, -23.90)	.03	3095.1	2917.6, 3272.5	-242.22 (-864.42, 379.99)	.44	3151.6	2835.6, 3467.7
Race/Ethnicity (District)								

© 2020 Turner L et al. *JAMA Network Open.*

≥66% White	0 [Reference]	--	3232.4	3067.2, 3397.6	0 [Reference]	--	3278.1	2968.3, 3587.8
≥50% Black	246.10 (-59.99, 552.19)	.11	3478.5	3211.0, 3746.0	292.15 (-653.02, 1237.33)	.54	3570.2	2624.7, 4515.7
≥50% Hispanic	-89.25 (-395.08, 216.58)	.56	3143.2	2923.3, 3363.0	-249.37 (-775.86, 277.13)	.35	3028.7	2701.1, 3356.2
Other	-153.82 (-393.28, 85.65)	.21	3078.6	2908.0, 3249.1	-225.96 (-645.90, 193.99)	.29	3052.1	2770.1, 3334.1
Child Poverty Rate (District)								
<20%	0 [Reference]	--	3193.8	3051.6, 3336.0	0 [Reference]	--	3103.2	2909.9, 3296.6
≥20%	-85.41 (-271.30, 100.47)	.36	3108.4	2978.1, 3238.7	182.96 (-89.88, 455.80)	.19	3286.2	3010.1, 3562.2
Region								
West	0 [Reference]	--	3273.5	3036.8, 3510.3	0 [Reference]	--	3396.9	2820.7, 3973.1
Midwest	46.15 (-269.95, 362.24)	.77	3319.7	3103.9, 3535.4	-227.81 (-815.22, 359.60)	.44	3169.1	2929.0, 3409.1
South	-127.52 (-408.46, 153.42)	.37	3146.0	3027.1, 3264.9	-179.26 (-838.28, 479.75)	.59	3217.6	2951.5, 3483.7
Northeast	-464.44 (-794.67, -134.22)	.006	2809.1	2532.9, 3085.3	-787.78 (-1458.21, -117.35)	.02	2609.1	2066.6, 3151.6

© 2020 Turner L et al. *JAMA Network Open.*

eTable 4. Multivariable Logistic Regression Model Examining the Association of State-Level Smart Snacks Policy With Student Consumption of Snacks Obtained at School Among 1959 Students

Variable	Odds Ratio (95% CI)	P Value	Adjusted Prevalence	95% CI
State Law Requires Smart Snacks Standards to Be Followed in All School Venues				
No	1 [Reference]	--	26.9	23.8, 30.0
Yes	0.82 (0.59, 1.15)	.25	23.3	18.3, 28.4
Student Grade (continuous)	1.06 (1.02, 1.11)	.007	--	
Student Gender				
Male	1 [Reference]	--	23.3	19.7, 27.0
Female	1.35 (1.03, 1.75)	.03	28.9	25.3, 32.6
Student Race/Ethnicity				
White, non-Hispanic	1 [Reference]	--	23.8	19.9, 27.8
Black, non-Hispanic	1.82 (1.22, 2.71)	.004	36.0	28.4, 43.7
Hispanic	1.13 (0.80, 1.61)	.48	26.1	21.1, 31.2
Other	1.08 (0.65, 1.77)	.77	25.2	17.7, 32.6
School Urbanicity				
Urban	1 [Reference]	--	27.0	18.9, 35.0
Suburban	0.93 (0.56, 1.53)	.77	25.6	21.7, 29.4
Rural	0.97 (0.60, 1.55)	.89	26.3	22.5, 30.1
School Size				
Large (≥1000 students)	1 [Reference]	--	27.4	22.3, 32.5
Medium (500 to 999 students)	0.90 (0.64, 1.28)	.57	25.5	21.4, 29.6
Small (< 500 students)	0.90 (0.57, 1.42)	.64	25.4	18.8, 31.9
Race/Ethnicity (District)				
≥66% White	1 [Reference]	--	28.4	23.6, 33.1
≥50% Black	0.94 (0.57, 1.56)	.82	27.3	19.1, 35.4
≥50% Hispanic	0.98 (0.51, 1.89)	.95	27.9	17.2, 38.7
Other	0.76 (0.53, 1.08)	.12	23.2	19.3, 27.0
Child Poverty Rate (District)				
<20%	1 [Reference]	--	26.6	22.3, 30.8
≥20%	0.93 (0.63, 1.37)	.70	25.2	20.5, 29.8
Region				
West	1 [Reference]	--	25.5	16.6, 34.4
Midwest	1.05 (0.60, 1.86)	.86	26.4	21.5, 31.4
South	1.08 (0.61, 1.92)	.79	27.0	22.8, 31.1
Northeast	0.89 (0.44, 1.80)	.74	23.3	14.8, 31.8

© 2020 Turner L et al. *JAMA Network Open.*
